# Genomic and Phenotypic Variability in *Neisseria gonorrhoeae* Antimicrobial Susceptibility, England

**DOI:** 10.3201/eid2603.190732

**Published:** 2020-03

**Authors:** Katy Town, Simon Harris, Leonor Sánchez-Busó, Michelle J. Cole, Rachel Pitt, Helen Fifer, Hamish Mohammed, Nigel Field, Gwenda Hughes

**Affiliations:** National Institute for Health Research, London, UK (K. Town, G. Hughes);; Public Health England, London (K. Town, M.J. Cole, R. Pitt, H. Fifer, H. Mohammed, G. Hughes);; University College London, London (K. Town, N. Field, G. Hughes);; Microbiotica Ltd, Cambridge, UK (S. Harris);; Wellcome Sanger Institute, Cambridge (S. Harris, L. Sánchez-Busó);; University of Oxford, Oxford, UK (L. Sánchez-Busó)

**Keywords:** Molecular epidemiology, whole-genome sequencing, Neisseria gonorrhoeae, gonorrhea, antimicrobial susceptibility, antimicrobial resistance, ceftriaxone, penA, bacteria, AMR, England

## Abstract

Antimicrobial resistance (AMR) in *Neisseria gonorrhoeae* is a global concern. Phylogenetic analyses resolve uncertainties regarding genetic relatedness of isolates with identical phenotypes and inform whether AMR is due to new mutations and clonal expansion or separate introductions by importation. We sequenced 1,277 isolates with associated epidemiologic and antimicrobial susceptibility data collected during 2013–2016 to investigate *N. gonorrhoeae* genomic variability in England. Comparing genetic markers and phenotypes for AMR, we identified 2 *N. gonorrhoeae* lineages with different antimicrobial susceptibility profiles and 3 clusters with elevated MICs for ceftriaxone, varying mutations in the *penA* allele, and different epidemiologic characteristics. Our results indicate *N. gonorrhoeae* with reduced antimicrobial susceptibility emerged independently and multiple times in different sexual networks in England, through new mutation or recombination events and by importation. Monitoring and control for AMR in *N. gonorrhoeae* should cover the entire population affected, rather than focusing on specific risk groups or locations.

Antimicrobial resistance (AMR) in *Neisseria gonorrhoeae* is a global concern and affects all classes of antimicrobial drugs used for treatment. Penicillin, ciprofloxacin, and cefixime were the recommended first-line antimicrobial drug therapies until AMR prevalence breached the World Health Organization (WHO) recommended threshold of >5% of local isolates demonstrating resistance; at that point, ceftriaxone became the preferred antimicrobial drug treatment (*1*). However, ceftriaxone resistance has been reported in many countries and frequently in East and Southeast Asia, probably because of poor antimicrobial stewardship (*2*). Ceftriaxone resistance has been linked to mutations in the *penA* gene, which has been reported in several continents, including North America and Europe (*3**–**5*).

To clarify the spread of AMR in *N. gonorrhoeae* and the population groups most at risk, surveillance programs and research studies often link phenotypic susceptibility data with data on the epidemiologic characteristics of cases (*6*,*7*). However, these analyses are limited because isolates with identical phenotypes might not be genetically related. Consequently, determining the extent to which AMR transmission is due to clonal dissemination or separate introductions is challenging and these data are essential to guide the public health response. 

Combining phenotypic and genomic data can help resolve uncertainties. Whole-genome sequencing (WGS) enables investigation of genetic determinants for AMR and how these are distributed in the pathogen population (*4*,*5*). WGS also can contribute evidence toward the development of rapid antimicrobial susceptibility tests to improve treatment decisions (*8*,*9*). However, few *N. gonorrhoeae* WGS studies have been conducted in England, and none include representative geographic coverage over time (*10**–**13*).

We investigated the genomic and phenotypic variability in *N. gonorrhoeae* antimicrobial susceptibility in England. We described the epidemiologic characteristics of genetically distinct clusters of infection with reduced susceptibility to cefixime, ceftriaxone, and azithromycin and resistance to ciprofloxacin and penicillin. We focused on *N. gonorrhoeae* with mutations in the *penA* allele, which contribute to reduced susceptibility to ceftriaxone. In addition, we assessed the genetic similarity of *N. gonorrhoeae* in England, Europe, and the United States to determine the extent to which international travel might influence the spread of AMR in *N. gonorrhoeae*.

## Methods

### Isolate Selection

We selected *N. gonorrhoeae* isolates from the archives of the Gonococcal Resistance to Antimicrobials Surveillance Programme (GRASP), a sentinel program implemented by Public Health England (PHE) in 2000. GRASP is designed to represent the gonococcal population in England (*14*) and includes clinical, sociodemographic, and behavioral data collected through the GUMCAD STI Surveillance System (https://www.gov.uk/guidance/gumcad-sti-surveillance-system) and directly from clinics. During a 3-month period each year, GRASP tests for antimicrobial susceptibility in consecutive isolates from all culture-positive *N. gonorrhoeae* cases identified in 26 sexual health clinics in England and Wales (*15*). GRASP collects ≈1,200–2,500 isolates annually for antimicrobial susceptibility testing (*16*). 

We selected isolates collected from 5 GRASP clinics, 2 in London and 3 in other geographically distinct areas in England: Birmingham, Bristol, and Liverpool. We chose these locations to obtain isolates from cases representing a broad range of sociodemographic and behavioral characteristics, including sex, sexual orientation, age, ethnicity, and HIV status (Appendix 1 Table 1). We sequenced all isolates collected during 2013–2016 by the 5 clinics and stored in the GRASP archive. We chose the most recent years of GRASP data to investigate prevailing trends and patterns. We did not include isolates from a 2015 outbreak of high-level azithromycin-resistant *N. gonorrhoeae* in the United Kingdom in this sampling frame because the isolates did not meet the eligibility criteria of our study. 

### Ethics Considerations

PHE has permission to process confidential patient data obtained by GRASP under Regulation 3 (Control of Patient Information) of the Health Service Regulations 2002. Information governance advice and ethics approval for this study were granted by the PHE Research Ethics and Governance Group.

### Antimicrobial Susceptibility Testing

GRASP tests isolates for antimicrobial susceptibility by using agar dilution methods, records MICs for antimicrobial drugs, and defines AMR by using European Committee on Antimicrobial Susceptibility Testing (EUCAST) breakpoints. For our study, we used data on MICs for ceftriaxone, azithromycin, cefixime, penicillin, and ciprofloxacin (*17*). In GRASP, epidemiologic data were linked to MICs for antimicrobial drugs for each isolate phenotype.

### Isolation and WGS

We retrieved selected isolates from the GRASP archive by culturing on nonselective gonococcus agar (Difco BBL GC II Agar Base [Becton, Dickinson and Company, https://www.bd.com] plus 1% Vitox [Oxoid, http://www.oxoid.com]). We extracted DNA from a subculture of a single colony of each isolate by using the automated QIAsymphony DNA Mini Kit (QIAGEN, https://www.qiagen.com). WGS was conducted at the Wellcome Sanger Institute (Cambridge, UK) by using the HiSeq X Ten system (Illumina, https://www.illumina.com) and processed in the routine Sanger WGS data management pipeline (Appendix 1).

### Data Sources from Europe and the United States

We compared the study sample with published WGS and associated metadata for *N. gonorrhoeae* isolates from international studies. The collection from Europe (European Nucleotide Archive (ENA; accession no. PRJEB9227) contained 1,054 isolates from the European Gonococcal Antimicrobial Surveillance Programme (Euro-GASP) (*5*). Isolates were collected in 2013 from 20 countries and included 106 isolates from England. We excluded 21% (22/106) that were duplicates of isolates in the study sample, leaving 948 isolates from Europe. The metadata for isolates from Europe included reporting country, antimicrobial susceptibility profile, MICs for ceftriaxone and cefixime, and the presence of the *penA*-34 allele. We grouped MICs to match the categories used in GRASP. 

Isolates from the United States were from 2 previous studies investigating the association between phenotype and genotype for AMR in *N. gonorrhoeae* (*4*,*9*). The collection from the United States contained 1,114 isolates collected during 2000–2013 (ENA accession nos. PRJEB2999 and PRJEB7904). The metadata for the isolates from the United States included antimicrobial susceptibility profiles; 270 had reduced susceptibility to cephalosporin (MIC >0.25 mg/L for cefixime or MIC >0.125 mg/L for ceftriaxone); 294 had reduced susceptibility to azithromycin (MIC >2 mg/L); and 594 were ciprofloxacin-resistant (MIC >1 mg/L). Metadata also included sexual orientation of case-patients and the presence of the *penA*-34 allele. We grouped MICs to match the categories used in GRASP.

### Phylogenetic Analysis

We created phylogenetic trees and removed genetic recombination events by using default settings in Gubbins version 2.4.0 (*18*), including 5 iterations and >3 base substitutions to identify a recombination event, and the RAxML (Geneious, https://www.geneious.com) or FastTree (*19*) tree building option (Appendix 1). We created 3 phylogenetic trees: isolates from England only, isolates from England and other countries in Europe, and isolates from England and the United States.

We identified known genetic markers of AMR, including mutations in the *penA* allele, by using ARIBA (*20*). We compared MICs to the genetic markers by using the ARIBA micplot module.

We identified the genotype of isolates in large and distinct clusters of *N. gonorrhoeae* with elevated MICs for ceftriaxone (MIC ≥0.015 mg/L) and cefixime (MIC ≥0.03 mg/L) from the phylogenetic trees. We compared the epidemiologic characteristics of cases in the clusters by using the χ^2^ or Fisher exact test.

### Statistical Analysis

We used univariate and multivariable analyses to assess differences in the epidemiologic characteristics and antimicrobial susceptibility of isolates between lineages identified in the phylogenetic tree. We analyzed the following explanatory variables: year and location the isolate was collected; case-patient information, including gender, sexual orientation, age, ethnicity, country of birth, whether they had a symptomatic *N. gonorrhoeae* infection or previous sexually transmitted infection (STI), HIV status, and the number of sexual partners they had in the United Kingdom or through travel-associated sexual partnerships <3 months before diagnosis; and isolate susceptibility data, including reduced susceptibility to ceftriaxone (MIC >0.015 mg/L), cefixime (MIC >0.03 mg/L), or azithromycin (MIC >0.25 mg/L); or resistance to penicillin (MIC >1 mg/L or β-lactamase positive) or ciprofloxacin (MIC >0.06 mg/L). We used elevated MIC thresholds for ceftriaxone, cefixime, and azithromycin to provide a robust sample size for regression analysis. 

We also explored the relationship between travel-associated sexual partnerships and reduced susceptibility to antimicrobial drugs by conducting univariate and multivariable analyses with reduced susceptibility or resistance as the outcome and travel-associated sexual partnerships as the primary explanatory variable. We considered CI of the odds ratio (OR) >1.0 and p<0.05 by χ^2^ test as statistically significant. 

We developed multivariable logistic regression models by using a forward approach and including only statistically significant variables associated with the outcome in the univariate model to control for possible confounding between variables. We used the likelihood ratio test to determine which explanatory variables should remain in the multivariable model by using p<0.05 as the threshold of statistical significance.

## Results

### Sample Description

Of the eligible isolates, we successfully sequenced 91% (1,277/1,407); the bacteria of the remaining 130 isolates were no longer viable for DNA extraction. For all antimicrobial drugs tested, the MIC distributions of the study isolates were similar to those of all GRASP isolates (Appendix 1 Table 2). We found that 3.6% of isolates were resistant to azithromycin (MIC >0.5 mg/L) and 2 isolates were highly resistant (MIC >256 mg/L); 0.6% were resistant to cefixime (MIC >0.125 mg/L), 36.3% to ciprofloxacin (MIC >0.06 mg/L), 16.6% to penicillin (MIC >1 mg/L), and none to ceftriaxone (MIC >0.125 mg/L). The MIC distribution of the isolates not sequenced was similar to the distribution of the sequenced isolates. Most (69%; 881/1,277) isolates were from genital infections, 23.4% (299/1,277) were from rectal infections, and 6.3% (80/1,277) were from pharyngeal infections. Overall, we identified 226 different sequence types (STs) by using *N. gonorrhoeae* multiantigen sequence typing (NG-MAST) (Appendix 1). We deposited novel sequences extracted for this study into ENA (accession no. ERP022090) and provide metadata (Appendix 2).

### *N. gonorrhoeae* Lineages Circulating in England

**Figure 1:**
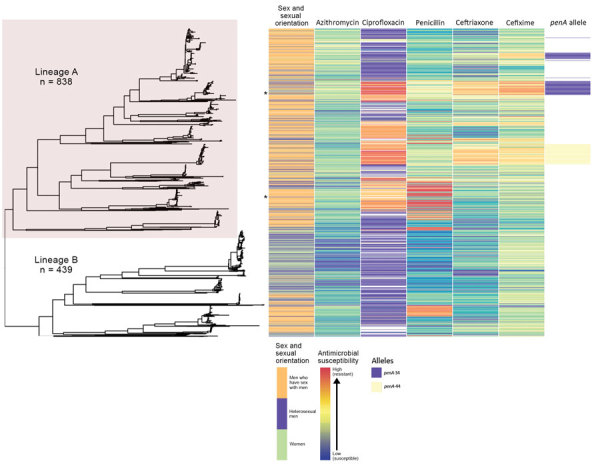
Phylogeny and antimicrobial susceptibility of *Neisseria gonorrhoeae* isolates from England, 2013–2016. Maximum-likelihood phylogeny with recombination events removed of all *N. gonorrhoeae* isolates annotated with gender and sexual orientation, antimicrobial susceptibility phenotype, and *penA* genotype. Asterisks represent location in tree of isolates with high-level azithromycin resistance (MIC >256 mg/L). Heterosexual men were those who reported sex with women exclusively.

We noted 2 distinct lineages in the phylogenetic tree (Figure 1). Compared with lineage B, lineage A was more likely to contain isolates from clinics in London than those outside of London (outside London:in London adjusted odds ratio [aOR] 1.74, 95% CI 1.27–2.67; p = 0.001). Lineage A also contained more isolates from persons >35 years of age than persons <24 years of age (aOR 1.68, 95% CI 1.16–2.40; p = 0.006). Asian ethnicity also was associated more frequently with isolates from lineage A compared with white ethnicity (aOR 1.86, 95% CI 1.01–3.45; p = 0.048) (Table 1). Lineage A was less likely to contain isolates from women (aOR 0.14, 95% CI 0.09–0.22; p<0.001) or men who reported having sex with women exclusively (MSW; aOR 0.33, 95% CI 0.23–0.47; p<0.001) compared with MSM. This lineage also was less likely to contain isolates from persons reporting black Caribbean ethnicity compared with persons reporting white ethnicity (aOR 0.49, 95% CI 0.32–0.76; p = 0.001). Lineage A was more likely to contain isolates from persons who reported a travel-associated sexual partnership compared with isolates from lineage B (crude odds ratio [cOR] 1.96, 95% CI 1.20–3.21; p = 0.006), but this association did not persist in the multivariable model (aOR 1.66, 95% CI 0.94–2.91; p = 0.078 adjusting for location, age, sex, sexual orientation, and ethnicity). However, isolates from persons who had a recent travel-associated sexual partnership were more likely to be resistant to ciprofloxacin (aOR 1.83, 95% CI 1.13–2.96; p = 0.015, adjusting for location, age, sex, sexual orientation, and ethnicity). We saw no statistically significant association between recent travel-associated sexual partnerships and *N. gonorrhoeae* with reduced susceptibility to ceftriaxone, cefixime, or azithromycin, or resistance to penicillin.

**Table 1:** Univariate and multivariable analyses comparing the epidemiologic characteristics of cases of *Neisseria gonorrhoeae* between 2 phylogenetic lineages, England*

Characteristics	Lineage, no.			Lineage A outcomes						
				Univariate				Multivariable		
	A	B		cOR	95% CI	p value		aOR	95% CI	p value
Total	838	439								
Year										
2013	220	106		Ref						
2014	210	123		0.82	0.60–1.13	0.234				
2015	260	107		1.17	0.85–1.62	0.339				
2016	148	103		**0.69**	**0.49**–**0.98**	**0.035**				
Clinic location										
Outside London	630	136		Ref				Ref		
London	463	109		**3.73**	**2.86**–**4.88**	**<0.001**		**1.74**	**1.27**–**2.67**	**0.001**
Sex and sexual orientation										
MSM	630	136		Ref				Ref		
MSW	150	154		**0.21**	**0.15**–**0.29**	**<0.001**		**0.33**	**0.23**–**0.47**	**<0.001**
F	57	149		**0.08**	**0.05**–**0.12**	**<0.001**		**0.14**	**0.09**–**0.22**	**<0.001**
Age, y										
≤24	188	196		Ref				Ref		
25–34	342	161		**2.21**	**1.67**–**2.93**	**<0.001**		1.14	0.83–1.59	0.413
≥35	308	82		**3.92**	**2.81**–**5.46**	**<0.001**		**1.68**	**1.16**–**2.40**	**0.006**
Ethnicity										
White	586	238		Ref				Ref		
Black Caribbean	51	81		**0.26**	**0.17**–**0.38**	**<0.001**		**0.49**	**0.32**–**0.76**	**0.001**
Black African	27	20		**0.55**	**0.30**–**1.00**	**0.046**		0.84	0.43–1.64	0.607
Black Other	6	4		0.61	0.17–2.18	0.442		0.57	0.14–2.37	0.441
Asian	57	17		1.36	0.78–2.39	0.280		**1.86**	**1.01**–**3.45**	**0.048**
Other	24	8		1.22	0.54–2.75	0.634		0.99	0.41–2.44	0.999
Mixed	62	43		**0.59**	**0.39**–**0.89**	**0.011**		0.82	0.51–1.32	0.413
Place of birth										
United Kingdom	473	309		Ref						
Not United Kingdom	305	102		**1.95**	**1.49**–**2.56**	**<0.001**				
Symptomatic infection										
No	219	119		Ref						
Yes	526	277		1.03	0.79–1.35	0.818				
New STI diagnosis <1 year, excluding HIV										
No or unknown	615	363		Ref						
Yes	223	75		**1.75**	**1.31**–**2.35**	**<0.001**				
HIV status										
Negative or unknown	653	398		Ref						
Positive	185	41		**2.75**	**1.91**–**3.96**	**<0.001**				
Number of partners in the United Kingdom <3 months of diagnosis										
0	27	20		Ref						
1	175	162		0.80	0.43–1.48	0.478				
≥2	304	167		1.35	0.73–2.48	0.335				
Travel-associated sexual partnerships <3 months of diagnosis										
No	442	325		Ref						
Yes	64	24		**1.96**	**1.20**–**3.21**	**0.006**				

*The multivariable model was adjusted for location, gender and sexual orientation, age, and ethnicity. Bold text indicates statistical significance (p<0.05 and 95% CI does not cross 1.0). aOR, adjusted odds ratio; cOR, crude odds ratio; MSM, men who have sex with men; MSW, men who reported sexual activity exclusively with women; Ref, referent; STI, sexually transmitted infection.

Isolates with reduced susceptibility to ceftriaxone, cefixime, or azithromycin or resistance to ciprofloxacin and penicillin were dispersed throughout the phylogenetic tree (Figure 1). However, compared with lineage B, isolates in lineage A were more likely to have higher MICs for ceftriaxone (aOR 15.4, 95% CI 8.50–27.8; p<0.001), cefixime (aOR 3.97, 95% CI 2.76–5.76; p<0.001), azithromycin (aOR 7.5, 95% CI 5.37–10.5; p<0.001), and penicillin (aOR 18.2, 95% CI 11.4–29.2; p<0.001) (Table 2).

**Table 2:** Association between antimicrobial susceptibility of *Neisseria gonorrhoeae* isolates and presence in lineage A of the phylogeny, England*

Susceptibility	Lineage A, no. isolates	Lineage B, no. isolates	aOR	95% CI	p value
Reduced					
Ceftriaxone, MIC ≥0.015 mg/L					
No	572	418	Referent	–	–
Yes	263	15	**15.4**	**8.50**–**27.8**	**<0.001**
Cefixime, MIC ≥0.03 mg/L					
No	544	370	Referent	–	–
Yes	291	63	**3.97**	**2.76**–**5.76**	**<0.001**
Azithromycin, MIC ≥0.25 mg/L					
No	328	367	Referent	–	–
Yes	507	66	**7.50**	**5.37**–**10.5**	**<0.001**
Resistant					
Penicillin, MIC >1 mg/L or β-lactamase positive					
No	671	378	Referent	–	–
Yes	164	55	1.33	0.92–1.93	0.134
Ciprofloxacin, MIC >0.06 mg/L					
No	400	408	Referent	–	–
Yes	435	25	**18.2**	**11.4**–**29.2**	**<0.001**

*Each model adjusted for location inside or outside of London, and patient age, sexual orientation, and ethnicity. Nine isolates did not have MIC data. Bold text indicates statistical significance, i.e., p<0.05 and 95% CI does not cross 1. aOR, adjusted odds ratio.

### Distribution of *penA* Alleles across the Phylogeny

**Figure 2:**
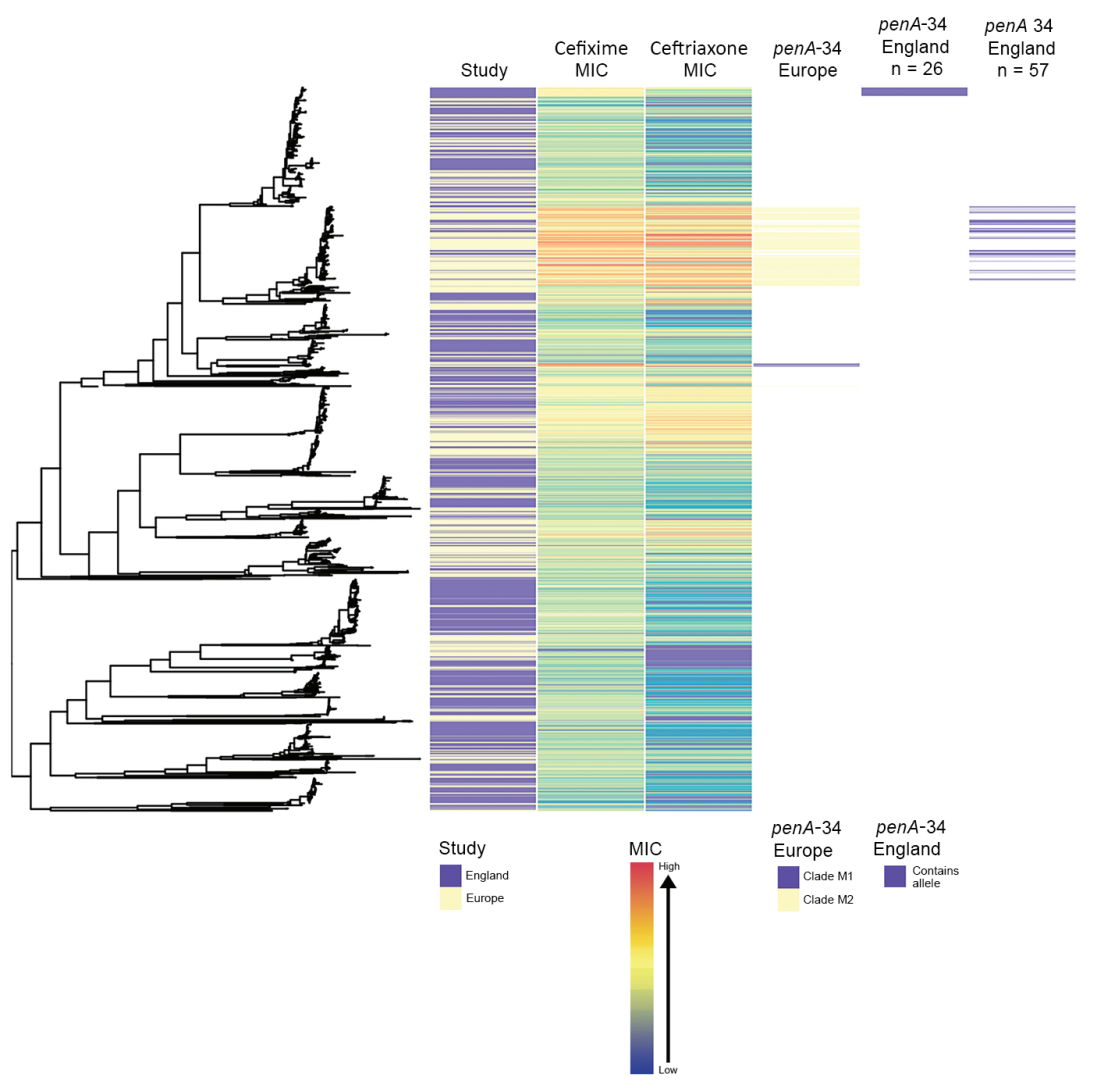
Phylogenetic tree of *Neisseria gonorrhoeae* isolates from England and other countries in Europe in a study of antimicrobial susceptibility, 2013–2016, including metadata for study type, MICs for ceftriaxone and cefixime, and presence of *penA*-34 alleles. We sequenced 1,277 isolates; 948 isolates were from other countries in Europe. The *penA*-34 clades from Europe are labeled M1 and M2, as noted by Harris et al. (*5*).

**Figure 3:**
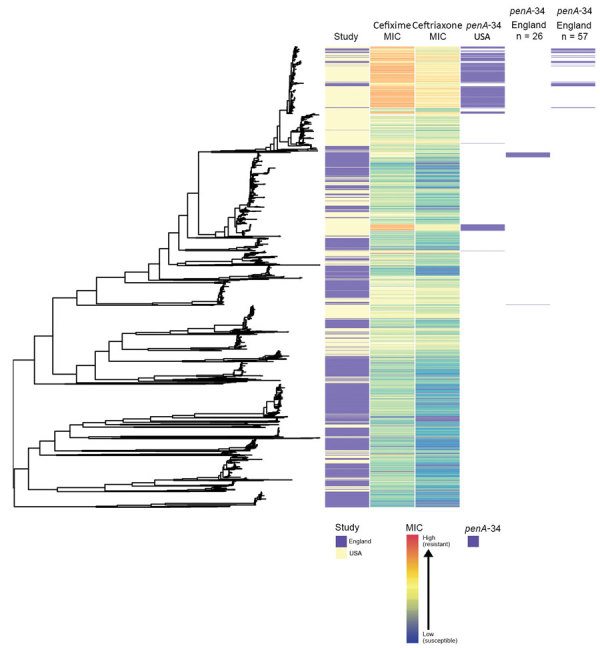
Phylogenetic tree of *Neisseria gonorrhoeae* isolates from England and the United States in a study of antimicrobial susceptibility, 2013–2016, including metadata for study type, MICs for ceftriaxone and cefixime, and presence of *pen*A-34 alleles. We sequenced 1,277 isolates; 1,114 isolates were from the United States.

Overall, we identified 32 different known mutations in 8 genes associated with resistance to ceftriaxone, cefixime, azithromycin, ciprofloxacin, or penicillin. For all antimicrobial drugs, we noted isolates with the same combination of genotypic markers of resistance but differing phenotypic MICs (Appendix 1 Figures 1–5).

The larger, distinct clusters with elevated MICs for ceftriaxone and cefixime contained the *penA*-34 allele and the *penA*-44 allele (Figure 1). All isolates with the *penA*-34 allele (n = 86) had a MIC of >0.015 mg/L for cefixime and 67 had a MIC of >0.015 mg/L for ceftriaxone. Most (81/84; 96%) isolates with the *penA*-44 allele had a MIC of >0.015 mg/L for cefixime, a MIC of >0.015 mg/L for ceftriaxone (83/84; 98%), or both (81/84; 96%). 

The 2 largest clusters with the *penA*-34 allele (cluster 1, n = 57; cluster 2, n = 26) were genetically distinct from each other and isolates in the 2 groups had statistically significant differences by year, clinic, sexual orientation, and HIV status (Table 3). Most (81%; 21/26) isolates in cluster 2 were from London in 2014–2015, and most (67%; 38/57) in cluster 1 were from outside London but distributed across all 4 years of the study. Most (96%; 25/26) isolates in cluster 2 were from MSM, whereas cluster 1 was more mixed and composed of isolates from women (21%; 12/57), MSW (37%; 21/57), and MSM (42%; 24/57). Cluster 2 had a higher percentage of persons living with HIV (35%; 9/26) than did cluster 1 (7%; 4/57). Most (82%; 69/84) isolates with the *penA*-44 allele were from MSM, persisted over all 4 years of the study, and were found both inside and outside of London. The characteristics of isolates with the *penA*-44 allele were more similar to the characteristics of isolates in cluster 2 of the *penA*-34 group than to isolates in cluster 1 (Appendix 1 Table 3).

**Table 3:** Epidemiologic characteristics of patients from whom *Neisseria gonorrhoeae* isolates were collected in the 2 largest *penA*-34 clusters, England*

Characteristics	Total	Cluster 1, n = 57, no. (%)	Cluster 2, n = 26, no. (%)	p value†
Year				
2013	26	26 (45.6)	0	**<0.001**‡
2014	29	14 (24.6)	15 (57.7)	
2015	20	10 (17.5)	10 (38.5)	
2016	8	7 (12.3)	1 (3.8)	
Sex and sexual orientation				
MSM	49	24 (42.1)	25 (96.2)	**<0.001**‡
MSW	21	21 (36.8)	0	
F	13	12 (21.1)	1 (3.8)	
Clinic location				
Outside London	43	38 (66.7)	5 (19.2)	**<0.001**‡
London	40	19 (33.3)	21 (80.8)	
Age, y				
≤24	28	23 (40.4)	5 (19.2)	0.081
25–34	29	20 (35.1)	9 (34.6)	
≥35	26	14 (24.6)	12 (46.2)	
Ethnicity				
White	59	37 (68.5)	22 (84.6)	0.408‡
Black Caribbean	6	6 (11.1)	0	
Black Other	2	2 (3.7)	0	
Asian	5	4 (7.4)	1 (3.8)	
Other	4	2 (3.7)	2 (7.7)	
Mixed	4	3 (5.6)	1 (3.8)	
Place of birth				
United Kingdom	40	30 (52.6)	10 (38.5)	0.262‡
Not United Kingdom	40	26 (45.6)	14 (53.9)	
Unknown	3	1 (1.7)	2 (7.7)	
Symptomatic infection				
No	28	13 (24.5)	15 (57.7)	**0.004**
Yes	51	40 (75.5)	11 (42.3)	
New STI diagnosed <1 year, excluding HIV				
No	68	51 (89.5)	17 (65.4)	**0.013**‡
Yes	15	6 (10.5)	9 (34.6)	
HIV status				
Negative or unknown	70	53 (93.0)	17 (65.4)	**0.003**‡
Positive	13	4 (7.0)	9 (34.6)	
Number of sexual partners in the United Kingdom <3 mo of *N. gonorrhea* diagnosis				
0	6	6 (13.3)	0	0.675‡
1	19	16 (35.6)	3 (33.3)	
≥2	29	23 (51.1)	6 (66.7)	
Travel-associated sexual partnership				
No	43	34 (75.6)	9 (100)	0.178‡
Yes	11	11 (24.4)	0	

*Bold text indicates statistical significance (i.e., p<0.05 and 95% CI does not cross 1). MSM, men who have sex with men; MSW, men who reported sexual activity exclusively with women. †Calculated using χ^2^ test, except where noted.  ‡Calculated using Fisher exact test.

### Comparison of Isolates from England, Europe, and the United States

Isolates from England were genetically interspersed with isolates from other countries in Europe (Figure 2) or the United States (Figure 3), although some large clades of isolates came only from England or the United States. Isolates in cluster 1 with the *penA*-34 allele in England were clustered with isolates from Europe and the United States (Figures 2–3). Isolates with the *penA*-34 allele in cluster 2 from England that were only found after 2013 were not related genetically to isolates from the United States or from other countries in Europe.

## Discussion

We conducted a large study on genomic variability of antimicrobial susceptibility in *N. gonorrhoeae* in England. We sampled isolates from geographically dispersed clinics in England, and our data likely represent patterns at a national level. Our data suggest that *N. gonorrhoeae* with reduced susceptibility to antimicrobial drugs, including ceftriaxone, has emerged in England through novel mutation and recombination events, repeated introduction from overseas, clonal expansion, or a combination of these. 

We observed 3 distinct clusters with 2 different *penA* alleles and reduced susceptibility to ceftriaxone and cefixime and found patients in each cluster with differing epidemiologic characteristics. The genetic similarity of isolates from England, Europe, and the United States is consistent with global dissemination of *N. gonorrhoeae* concerning genotypic and phenotypic features. Our data highlight the potential influence of travel-associated sexual partnerships in AMR transmission.

As seen in other *N. gonorrhoeae* studies, the high frequency of DNA recombination requires computational strategies to use single-nucleotide polymorphism differences arising through mutation rather than recombination. We identified and removed recombinant DNA, but some likely remained, which might lead to incorrect inferences about relatedness for some isolates.

We found 2 distinct lineages of *N. gonorrhoeae* with different antimicrobial susceptibility profiles circulating in England. The larger lineage contained isolates with elevated MICs or resistance to all 5 antimicrobial drugs tested, consistent with findings from recent studies in Europe and globally (*5*,*21*). The authors of those studies hypothesized that differing susceptibility profiles of the 2 lineages were associated with different sexual orientation networks, but neither study had complete data on sexual orientation to support the hypothesis. Our study includes sexual orientation data for 99% of cases. Our findings strongly support the hypothesis that MSM are more frequently infected with *N. gonorrhoeae* strains with reduced susceptibility to antimicrobials, whereas MSW and women are more frequently infected with the more susceptible lineage. Nevertheless, many MSW were infected with *N. gonorrhoeae* with reduced susceptibility to antimicrobial drugs and MSM were infected by antimicrobial susceptible *N. gonorrhoeae*, and we noted intralineage variation by sexual orientation. MSM have more bacterial STI diagnoses and greater exposure to antimicrobial drugs, thereby increasing selection pressures for AMR, a hypothesis supported by mathematical models (*21**–**24*). Resistant strains also might persist in the absence of selective pressure because the organism’s biologic fitness is unaffected or compensatory mutations mitigate a deleterious effect (*25**–**27*).

By combining WGS, epidemiologic, and phenotypic data, we found that reduced susceptibility to ceftriaxone and cefixime emerged repeatedly in separate sexual networks in England. Without WGS data, we would have grouped all *penA-*34 samples from MSM together. Likewise, if we restricted sequencing to the *penA* gene, we would not have identified unique clusters with the same *penA*-34 allele.

The large group of isolates in England with the *penA*-34 allele clustered with isolates from Europe and the United States that had the same allele. Some of the *penA*-34 allele isolates belonged to the NG-MAST 1407 lineage, a widely disseminated clone associated with elevated MICs for ceftriaxone and cefixime and the catalyst for changing national treatment guidelines in the United Kingdom from cefixime as first-line therapy to ceftriaxone in 2011 (*6*,*22*). Therefore, the larger *penA*-34 group probably represents clonal spread of a previously identified endemic strain of *N. gonorrhoeae*; the smaller *penA*-34 group represents a new strain emerging in a different sexual network largely comprising MSM in London with a history of STIs, including HIV. Consequently, restricting public health resources that measure, prevent, and control AMR in *N. gonorrhoeae* to specific risk groups or geographic locations could be ineffective because AMR appears to emerge independently in different sexual networks and locations.

We found some evidence for importation of AMR. Isolates from persons who recently had a travel-associated sexual partnership were more likely to be infected with *N. gonorrhoeae* that was resistant to ciprofloxacin. Although de novo development of high-level resistance to azithromycin in the United Kingdom has been described, some studies have concluded that importation events probably initiate AMR spread in countries with low population prevalence, such as England (*11*,*27*,*28*). The success of antimicrobial stewardship policies and compliance with treatment guidelines that aim to curtail AMR in the endemic gonococcal population in England could be undermined by the importation and subsequent spread of resistant isolates. These data support the importance of promoting STI prevention messages and testing to international travelers, particularly those visiting countries where AMR *N. gonorrhoeae* is endemic. Quantifying the relationship between *N. gonorrhoeae* circulating in England and internationally also could help parameterize mathematical models exploring the relative contribution of importation and de novo development on AMR prevalence and distribution.

Rapid molecular tests for genetic markers that are highly predictive of an antimicrobial susceptibility phenotype could lead to more effective use of antimicrobials. Tests detecting markers of ciprofloxacin and cephalosporin antimicrobial susceptibility are already in development (*8*,*29*,*30*). However, as found in our study and elsewhere, the association between genotype and phenotype is much stronger for ciprofloxacin resistance than for cephalosporin resistance (*4*,*31*,*32*). Most rapid tests for cephalosporin resistance focus on detecting mutations in the *penA* allele, but reduced susceptibility to cephalosporins also can be caused by mutations in the semimosaic *penA* allele, *penA*-35, the nonmosaic *penA* allele, *penA*-44, or even in the *mtrR* and *penB* genes (*30*,*33*).

Molecular tests that focus on the presence or absence of 1 mutation without considering the additive effect of multiple mutations could be insufficient for detecting resistance and predicting treatment failure. Epistasis, in which phenotypic resistance is dependent on complex interactions of multiple mutant genes, is known to occur in *N. gonorrhoeae* (*22*,*31*,*34*). In our study, the antimicrobial susceptibility of isolates with identical genetic markers of resistance varied by >2 doubling dilutions, and most isolates with resistance markers were sensitive. Nonetheless, the presence of 1 mutation that belongs to a complex of mutations required for resistance indicates the potential for phenotypic resistance to develop. Clinicians could prioritize patients infected with these strains for a test of cure or consider use of alternative antimicrobial drugs unaffected by the resistance marker. In any event, mathematical modeling studies have shown that molecular tests should only be implemented if they are highly sensitive; otherwise, they could accelerate the spread of AMR (*23*). Elucidation of the mechanisms and genomic markers of cephalosporin resistance is needed and can be achieved through a combination of microbiologic and genomic studies, including genome wide association studies. WGS cannot replace phenotypic testing for all antimicrobial susceptibility because it can only detect known mutations associated with resistance, and novel mutations associated with resistance develop constantly in *N. gonorrhoeae* (*31*).

In conclusion, phylogenetic analyses with WGS data revealed transmission patterns of *N. gonorrhoeae* with reduced susceptibility in England that would not have been identified by using only epidemiologic and phenotypic data. Reduced susceptibility to antimicrobial drugs likely has emerged and spread independently in different sexual networks in England through multiple de novo mutation and recombination events and through some repeated importation by persons who have travel-associated sexual partnerships. Consequently, public health actions to limit dissemination of AMR in England should aim to reduce risk behaviors that support *N. gonorrhoeae* transmission and encompass the diffuse distribution and epidemiologic diversity of the population groups affected.

Appendix 1 Additional information on genomic and phenotypic variability in *Neisseria gonorrhoeae* antimicrobial susceptibility, England.

Appendix 2 Metadata on 1,277 novel sequences extracted for a study on genomic and phenotypic variability of antimicrobial-susceptible *Neisseria gonorrhoeae*, England.
