# Supplementary material for: Genomic and Phenotypic Variability in Neisseria gonorrhoeae Antimicrobial Susceptibility, England
Source: Emerg Infect Dis. 2020 Mar;26(3):505–15. doi: 10.3201/eid2603.190732 (PMC7045833; doi:10.3201/eid2603.190732)
Supplement: Appendix 1 — Additional information on genomic and phenotypic variability in Neisseria gonorrhoeae antimicrobial susceptibility, England. [file 19-0732-Techapp-s1.pdf]

Article DOI: https://doi.org/10.3201/eid2603.190732

Genomic and Phenotypic Variability in *Neisseria gonorrhoeae* Antimicrobial Susceptibility, England

Appendix

This research was undertaken as part of the National Institute for Health Research Health Protection Research Unit (NIHR HPRU) in Blood-Borne and Sexually Transmitted Infections at University College London, England, UK.

Methods

Antimicrobial Susceptibility Testing External Quality Assurance

Quality assurance of antimicrobial susceptibility testing was performed by including 12 control strains, WHO A, WHO D, WHO E, WHO J, WHO K, WHO L, TR01, 1336, 1339, A02, A24, and QA07–09. In addition, we participated in external quality assurance (EQA) exchanges with the national reference laboratories of Scotland and Belgium, and in the Euro-GASP EQA.

Antimicrobial Resistance Definitions

The Gonococcal Resistance to Antimicrobials Surveillance Programme (GRASP) uses European Committee on Antimicrobial Susceptibility Testing (EUCAST) breakpoints to define antimicrobial resistance as follows: ceftriaxone, MIC >0.125 mg/L; cefixime, MIC >0.125 mg/L; azithromycin, MIC >0.5 mg/L; ciprofloxacin, MIC >0.06 mg/L; penicillin, MIC >1 mg/L.

Whole-Genome Sequencing Methods

Whole-genome sequencing (WGS) was conducted at the Wellcome Sanger Institute by using the HiSeq X Ten system (Illumina, http://www.illumina.com), and put through the routine Sanger WGS data management pipeline (*1*). The following measures were used to assess the quality of the WGS data for each isolate included in the phylogenetic analyses: a quality score >30 for the nucleotides called during the sequencing process, the majority of raw reads identified as *N. gonorrhoeae* when cross-referenced to a public database of pathogen genomes by using Kraken (*2*), the assembly length similar to the *N. gonorrhoeae* reference genome FA1090 (*3*),

2,153,922 nt, the assembly guanine and cytosine content (53%) similar to the *N. gonorrhoeae* reference genome FA1090, and >90% of the reference genome covered by reads.

After passing quality control, the raw reads were aligned to the reference genome FA1090 to create a consensus whole-genome sequence for each isolate. We used the Burrows-Wheeler Aligner Maximal Exact Match (BWA-MEM) algorithm with the option to flag duplicate shorter reads that match as secondary for removal (option M) (*4*). The Sequence Alignment/Map (SAM) (*5*) file output was converted into a Binary Alignment/Map (BAM) file using SAMTools to reduce the size of the file for faster computer processing (*5*). The Genome Analysis Toolkit (GATK) was used to realign indels, which helps the process of identifying single-nucleotide polymorphisms (SNPs) (*6*). SAMTools mpileup was used to identify the variant nucleotides identified in each read and the haploid option of Binary Call Format (BCF) tools from SAMTools filtered this information to select the variant nucleotides based on the following conditions: the minimum base call quality was $\geq$50 (quality of the base was previously determined using the Phred score system in SAMTools); the minimum mapping quality score by BWA-MEM was 20; $\geq$8 reads have the same variant and $\geq$3 are from each strand direction, forward and back; and the specific variant called is the same in 80% of the reads used. The consensus sequence for each isolate was compiled into 1 multiple FASTA file and used for the analyses.

Phylogenetic Tree Construction for Isolates from England

Gubbins version 2.4.0 (*7*) was used with the default settings (5 iterations and $\geq$3 base substitutions to identify a recombination event) and the tree building option Randomized Axelerated Maximum Likelihood (RAxML) version 8.2.8 (*8*) to create the phylogenetic tree with recombination events removed. Prior to this, the *opa* and *pil* genes, phages (*9*), and the Gonoccocal Genetic Island (GGI) (*10*) were manually removed from the alignment. The output phylogenetic tree was midpoint rooted, i.e., the root of the tree was placed half-way between the 2 isolates with the largest SNP difference, using Figtree version 1.4.3 (hhttp://tree.bio.ad.uk/software/figtree), and the branches were ladderized, i.e., branches were rotated so that they were ordered by increasing clade size at each node to aid visualization. Statistical support for the structure of the phylogenetic tree was assessed by using the Booster program (*11*). First, the phylogenetic tree was recreated by using Gubbins and RAxML with the bootstrap option to create 100 trees and input into Booster along with the reference tree used for

analysis. Booster was used to calculate the transfer bootstrap expectation (TBE), a value that quantifies the presence of each branch at a particular position in the bootstrap trees. A value of 1 indicates that the branch is in all bootstrap trees and a value of 0 indicates that the bootstrap trees are random. Statistical support for the phylogenetic tree was high: 79% (1,014/1,276) of nodes had a TBE value >70%.

Phylogenetic Tree Construction for Isolates from England, Europe, and the United States

For each international dataset, the consensus sequences were combined with the study consensus sequences and the mobile and repetitive elements removed. Phylogenetic trees with genetic recombination events removed were created by using Gubbins version 2.4.0 (*9*) with the default settings (5 iterations and ≥3 base substitutions to identify a recombination event) and the tree building option FastTree version 2.1.4 (*12*), which also uses a heuristic approach to find the tree with the maximum likelihood of producing the data given the model. FastTree has been shown to produce similar phylogenetic trees as RAxML methods but can analyze larger datasets within 1 day (*13*).

Results

N. gonorrhoeae Multiantigen Sequence Typing (NG-MAST) Data

The most common sequence types (STs) were ST51 (8.8%; 113/1,277), ST2992 (5%; 67/1,277), ST292 (3%; 45/1,277). By NG-MAST, the 2 isolates highly resistant to azithromycin had ST649 and ST13124, which are not the same STs seen in the United Kingdom during a 2015 outbreak of high-level azithromycin–resistant *N. gonorrhoeae*.

The largest *penA*-34 cluster contained 22 different previously reported STs and 4 novel STs. The 3 most frequently found STs were ST1407 (14/57; 25%), ST3169 (6/57; 10%), and ST8953 (5/57; 9%). The smaller *penA*-34 cluster primarily contained isolates with ST4244 (21/26; 81%). The remaining isolates were ST11084 (n = 2), ST3808 (n = 1), ST9897 (n = 1), and 1 isolate had a previously unidentified ST by NG-MAST. The *penA*-44 cluster contained 16 different STs, 30% (25/84) were ST2400, 21% (18/84) were ST10149, 17% (14/84) were ST6360, and 5 isolates had an ST that had not been identified previously.

References

1. Page AJ, De Silva N, Hunt M, Quail MA, Parkhill J, Harris SR, et al. Robust high-throughput prokaryote de novo assembly and improvement pipeline for Illumina data. Microb Genom. 2016;2:e000083. PubMed https://doi.org/10.1099/mgen.0.000083

2. Wood DE, Salzberg SL. Kraken: ultrafast metagenomic sequence classification using exact alignments. Genome Biol. 2014;15:R46. PubMed https://doi.org/10.1186/gb-2014-15-3-r46

3. National Center for Biotechnology Information. Genome assembly and annotation report: *Neisseria gonorrhoeae* FA 1090. [accessed 2018 Nov. 11] https://www.ncbi.nlm.nih.gov/genome/864?genome_assembly_id=300414

4. Li H. Aligning sequence reads, clone sequences and assembly contigs with BWA-MEM. arXiv 2013; 1303(3997). https://arxiv.org/abs/1303.3997

5. Li H, Handsaker B, Wysoker A, Fennell T, Ruan J, Homer N, et al.; 1000 Genome Project Data Processing Subgroup. The Sequence Alignment/Map format and SAMtools. Bioinformatics. 2009;25:2078–9. PubMed https://doi.org/10.1093/bioinformatics/btp352

6. McKenna A, Hanna M, Banks E, Sivachenko A, Cibulskis K, Kernytsky A, et al. The Genome Analysis Toolkit: a MapReduce framework for analyzing next-generation DNA sequencing data. Genome Res. 2010;20:1297–303. PubMed https://doi.org/10.1101/gr.107524.110

7. Croucher NJ, Page AJ, Connor TR, Delaney AJ, Keane JA, Bentley SD, et al. Rapid phylogenetic analysis of large samples of recombinant bacterial whole genome sequences using Gubbins. Nucleic Acids Res. 2015;43:e15. PubMed https://doi.org/10.1093/nar/gku1196

8. Stamatakis A. RAxML version 8: a tool for phylogenetic analysis and post-analysis of large phylogenies. Bioinformatics. 2014;30:1312–3. PubMed https://doi.org/10.1093/bioinformatics/btu033

9. Piekarowicz A, Kłyz A, Majchrzak M, Adamczyk-Popławska M, Maugel TK, Stein DC. Characterization of the dsDNA prophage sequences in the genome of Neisseria gonorrhoeae and visualization of productive bacteriophage. BMC Microbiol. 2007;7:66. PubMed https://doi.org/10.1186/1471-2180-7-66

10. Harrison OB, Clemence M, Dillard JP, Tang CM, Trees D, Grad YH, et al. Genomic analyses of *Neisseria gonorrhoeae* reveal an association of the gonococcal genetic island with antimicrobial resistance. J Infect. 2016;73:578–87. PubMed https://doi.org/10.1016/j.jinf.2016.08.010

11. Lemoine F, Domelevo Entfellner JB, Wilkinson E, Correia D, Dávila Felipe M, De Oliveira T, et al. Renewing Felsenstein's phylogenetic bootstrap in the era of big data. Nature. 2018;556:452–6. PubMed https://doi.org/10.1038/s41586-018-0043-0

12. Price MN, Dehal PS, Arkin AP. FastTree: computing large minimum evolution trees with profiles instead of a distance matrix. Mol Biol Evol. 2009;26:1641–50. PubMed http://dx.doi.org/10.1093/molbev/msp077

13. Lees JA, Kendall M, Parkhill J, Colijn C, Bentley SD, Harris SR. Evaluation of phylogenetic reconstruction methods using bacterial whole genomes: a simulation based study. Wellcome Open Res. 2018;3:33. PubMed https://doi.org/10.12688/wellcomeopenres.14265.1

Appendix Table 1. Epidemiologic characteristics of the study sample compared to all gonorrhea cases diagnosed in England, 2013–2016

Characteristics	Study sample, no. (%)	England, no. (%)	p value*
Total	1,267 (100.0)	146,369 (100.0)	
Year			
2013	326 (25.5)	31,213 (21.3)	**<0.001**
2014	333 (26.1)	37,178 (25.4)	0.580
2015	367 (28.7)	41,396 (28.3)	0.718
2016	251 (19.7)	36,582 (25.0)	**<0.001**
Clinic location			
London	527 (44.8)	72,809 (49.7)	**<0.001**
Outside London	705 (55.2)	73,560 (50.2)	**<0.001**
Gender and sexual orientation†			
MSM	766 (60.0)	72,660 (49.6)	**<0.001**
MSW	304 (23.8)	34,330 (23.5)	0.768
F	206 (16.1)	36,178 (24.7)	**<0.001**
Missing	1 (<0.1)	3,201 (2.2)	N/A
Age, y			
≤24	384 (30.1)	55,029 (37.6)	**<0.001**
25-34	503 (39.4)	54,143 (37.0)	0.077
≥35	390 (30.5)	37,197 (25.4)	**<0.001**
Ethnicity			
White	824 (64.5)	104,028 (71.1)	**<0.001**
Black Caribbean	132 (10.3)	8,280 (5.7)	**<0.001**
Black African	47 (3.7)	5,858 (4.0)	0.559
Black, other	10 (0.8)	3,238 (2.2)	**<0.001**
Asian	74 (5.8)	5,750 (3.9)	**<0.026**
Other	32 2.5)	4,747 (3.2)	0.138
Mixed	105 (8.2)	8,614 (5.9)	**<0.001**
Missing	53 (4.2)	5,815 (4.0)	0.747
Place of birth			
United Kingdom	782 (61.2)	96,189 (65.7)	**<0.001**
Not United Kingdom	407 (31.9)	38,334 (26.2)	**<0.001**
Missing	88 (6.8)	11,846 (8.1)	0.117
New STI diagnosed ≤1 year, excluding HIV			
N	1,015 (79.5)	117,493 (80.3)	0.481
Y	262 (20.5)	28,876 (19.7)	0.481
HIV status			
Negative or unknown	1,051 (82.3)	130,198 (89.0)	**<0.001**
Positive	226 (17.7)	16,171 (11.0)	**<0.001**

*p value calculated by using 2 sample proportions z-test. Bold text indicates statistical significance.
†MSM, men who report having sex with men; MSW, men who report having sex with women exclusively.

Appendix Table 2. Phenotypic antimicrobial susceptibility profile of study sample and all GRASP isolates, 2013–2016*

MIC	Study sample, no. (%)†	GRASP, no. (%)	p value†	Study sample as a % of GRASP
Total	1,267 (100.0)	6,184 (100.0)		20.5
Ceftriaxone (threshold for resistance, MIC >0.125 mg/L)				
<0.002	207 (16.3)	938 (15.2)	0.569	22.1
0.004	409 (32.3)	1,999 (32.3)		20.5
0.008	374 (29.5)	1,760 (28.5)		21.3
0.015	150 (11.8)	833 (13.5)		18.0
0.03	121 (9.6)	601 (9.7)		20.1
0.06	7 (0.6)	52 (0.8)		13.5
0.125	0	1 (<0.1)		0
Azithromycin (threshold for resistance, MIC >0.5 mg/L)				
<0.03	127 (10.0)	598 (9.7)	0.847	21.2
0.06	186 (14.7)	924 (14.9)		20.1
0.125	382 (30.1)	1,873 (30.3)		20.4
0.25	402 (31.7)	1,913 (30.9)		21.0
0.50	125 (9.9)	622 (10.1)		20.1
1.00	39 (3.1)	192 (3.1)		20.3
2.00	1 (0.1)	31 (0.5)		3.2
4.00	3 (0.2)	15 (0.2)		20.0
8.00	1 (0.1)	3 (0)		33.3
16.0	0	3 (0)		0
≥256	2 (0.2)	10 (0.2)		20.0
Cefixime (threshold for resistance, MIC >0.125 mg/L)				
0.002	37 (2.9)	200 (3.2)	0.795	18.5
0.004	62 (4.9)	305 (4.9)		20.3
0.008	331 (26.1)	1,691 (27.3)		19.6
0.015	484 (38.2)	2,187 (35.4)		22.1
0.03	174 (13.7)	882 (14.3)		19.7
0.06	144 (11.4)	729 (11.8)		19.8
0.125	28 (2.2)	140 (2.3)		20.0
0.25	8 (0.6)	46 (0.7)		17.4
0.50	0	4 (0.1)		0
Ciprofloxacin (2013–2015; threshold for resistance, MIC >0.06 mg/L)				
0.03	632 (49.9)	3,009 (48.7)	0.952	21.0
0.06	12 (0.9)	65 (1.1)		18.5
0.125	6 (0.5)	28 (0.5)		21.4
0.25	2 (0.2)	14 (0.2)		14.3
0.50	3 (0.2)	28 (0.5)		10.7
1.00	18 (1.4)	84 (1.4)		21.4
2.00	13 (1.0)	78 (1.3)		16.7
4.00	68 (5.4)	329 (5.3)		20.7
8.00	145 (11.4)	698 (11.3)		20.8
16.0	90 (7.1)	427 (6.9)		21.1
32.0	37 (2.9)	140 (2.3)		26.4
Ciprofloxacin (2016 breakpoint plates; threshold for resistance, MIC >0.06 mg/L)				
≤0.06	164 (12.9)	851 (13.8)	0.302	19.3
>0.06 and <0.50	5 (0.4)	13 (0.2)		38.5
≥0.50	73 (5.8)	420 (6.8)		17.4
Penicillin (threshold for resistance, MIC >1.0 mg/L)				
0.06	92 (7.3)	386 (6.2)	0.066	23.8
0.125	298 (23.5)	1,400 (22.6)		21.3
0.25	399 (31.5)	2,112 (34.2)		18.9
0.50	186 (14.7)	1,009 (16.3)		18.4
1.0	82 (6.5)	413 (6.7)		19.9
2.0	34 (2.7)	168 (2.7)		20.2
4.0	44 (3.5)	156 (2.5)		28.2
8.0	133 (10.5)	540 (8.7)		24.6

*GRASP, Gonococcal Resistance to Antimicrobials Surveillance Programme.
†Denominator is less than study sample because 10 samples from 2016 failed routine phenotypic testing so AMR data are unavailable.
‡p value from χ^2 test comparing distribution of MICs in the study sample to the GRASP sample.

Appendix Table 3. Epidemiologic characteristics of cases in *penA*-44 group compared to 2 *penA*-34 groups*

	penA-44 (n = 84)	*penA*-34 comparison, p value†	
Characteristics	No. (%)	Cluster 1 (n = 57)	Cluster 2 (n = 26)
Year			
2013	39 (46.4)	0.952	**<0.001**‡
2014	23 (27.4)		
2015	12 (14.3)		
2016	10 (11.9)		
Gender and sexual orientation§			
MSM	69 (82.1)	**<0.001**	0.116‡
MSW	11 (13.1)		
F	4 (4.8)		
Clinic location			
Outside London	38 (45.2)	**0.012**	**0.021**‡
London	46 (54.8)		
Age, y			
≤24	16 (19.0)	**0.021**	0.508
25–34	39 (46.4)		
≥35	29 (34.5)		
Ethnicity			
White	60 (72.3)	0.867‡	0.836‡
Black Caribbean	5 (6.0)		
Black African	1 (1.2)		
Black, other	1 (1.2)		
Asian	6 (7.2)		
Other	4 (4.8)		
Mixed	6 (7.2)		
Place of birth			
United Kingdom	56 (66.7)	0.201‡	**0.019**‡
Not United Kingdom	26 (31.0)		
Unknown	2 (2.4)		
Symptomatic infection			
N	19 (25.3)	0.917	**0.003**
Y	56 (74.7)		
New STI diagnosed <1 year, excluding HIV			
N	60 (71.4)	**0.01**	0.557
Y	24 (28.6)		
HIV status			
Negative or unknown	61 (72.6)	**0.003**	0.478
Positive	23 (27.4)		
Number of sexual partners in the United Kingdom ≤3 mo before diagnosis			
0	1 (1.9)	0.082	0.915
1	18 (34.0)		
≥2	34 (64.2)		
Travel-associated sexual partnership			
N	51 (96.2)	**0.003**	1.00‡
Y	2 (3.8)		

*Bold text indicates a statistically significant result, p<0.05 and CI does not cross 1. See Table 2 in the main manuscript for epidemiologic characteristics of isolates with *penA*-34 allele.
†p value calculated by using χ^2 test, except where indicated.
‡p value calculated by using Fisher exact test.
§MSM, men who reported sex with men; MSW, men who reported sex with women exclusively.

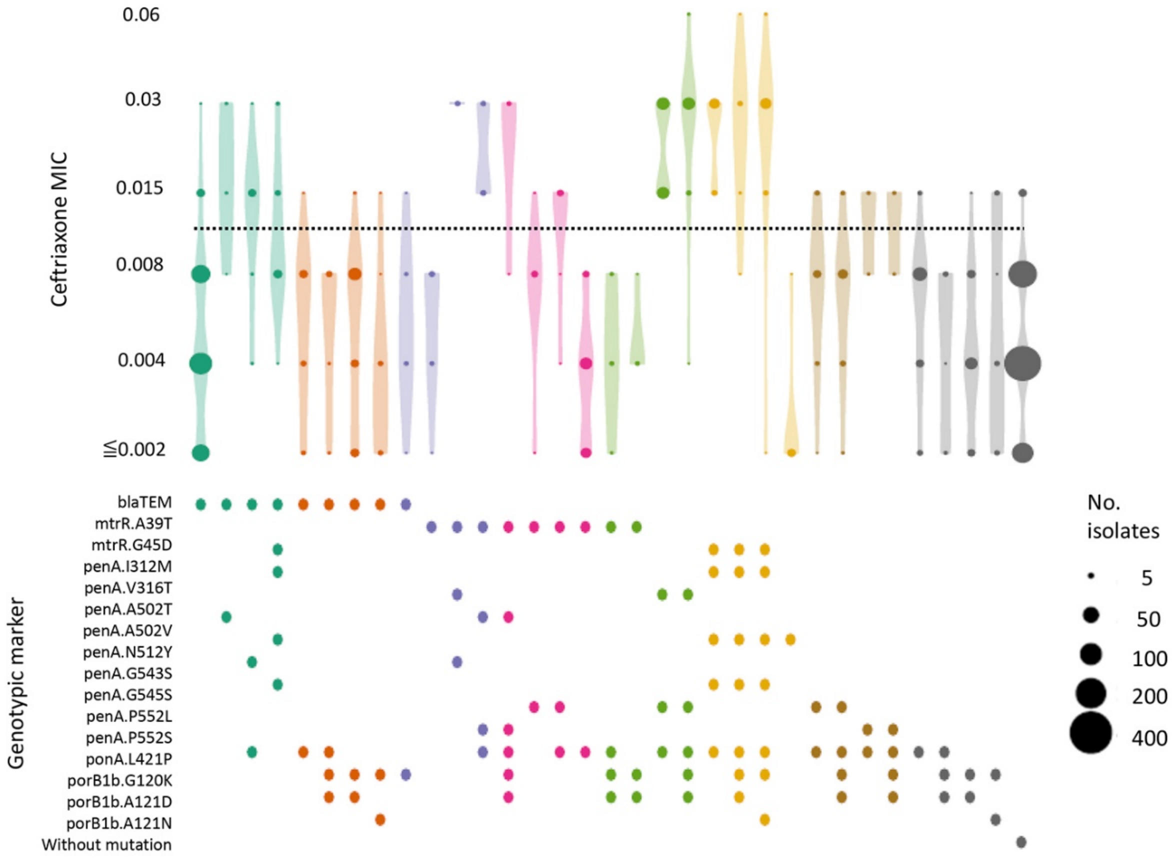

Appendix Figure 1. Genetic markers of antimicrobial resistance and MIC for ceftriaxone in a study of antimicrobial susceptibility of *Neisseria gonorrhoeae*, England, 2013–2016. Only marker combinations with ≥5 isolates included. Dotted line indicates reduced susceptibility threshold of MIC ≥0.015 mg/L. Colors are a visual aide to distinguish every 4 groups of genotypic markers and are not representative of any genotypic types. AMR, antimicrobial resistance; MIC, minimal inhibitory concentration.

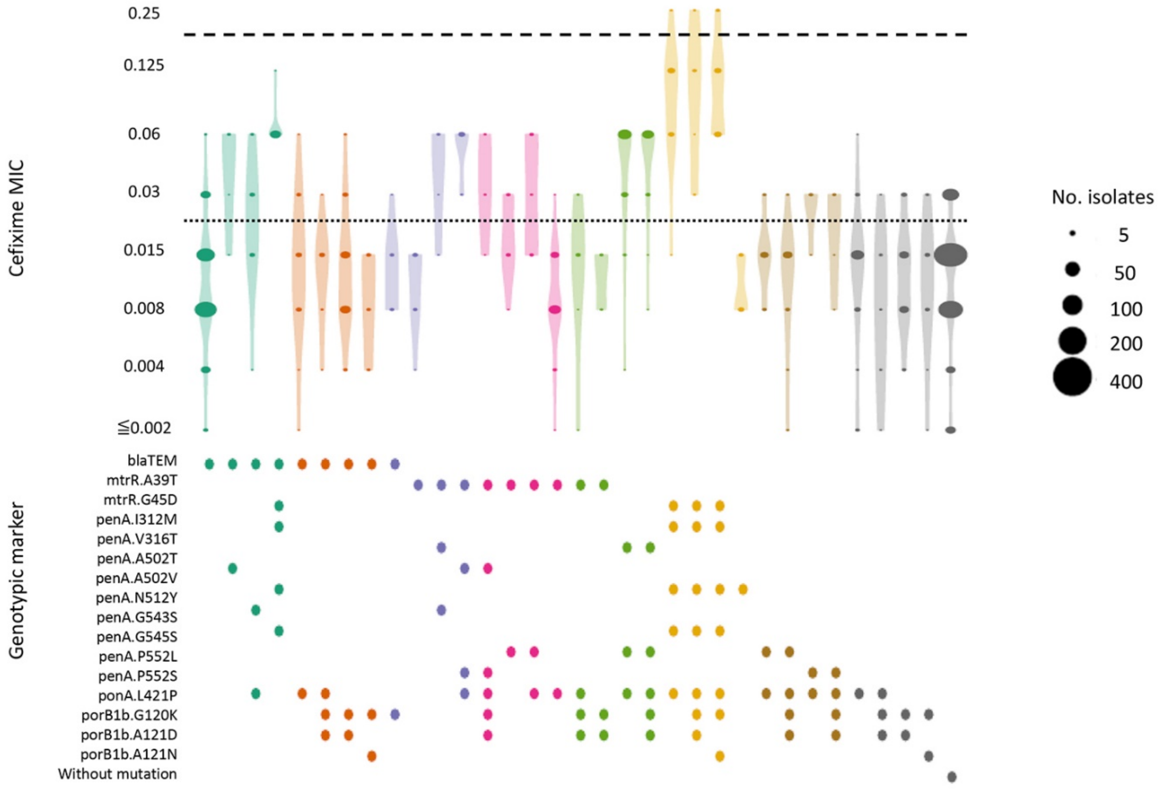

Appendix Figure 2. Genetic markers of antimicrobial resistance and MIC for cefixime in a study of antimicrobial susceptibility of *Neisseria gonorrhoeae*, England, 2013–2016. Only marker combinations with >5 isolates included. Dotted line indicates reduced susceptibility threshold of MIC >0.015 mg/L. Dashed line indicates resistance threshold of MIC >0.125 mg/L. The colors are a visual aide to distinguish every 4 groups of genotypic markers and are not representative of any genotypic types. AMR, antimicrobial resistance; MIC, minimal inhibitory concentration.

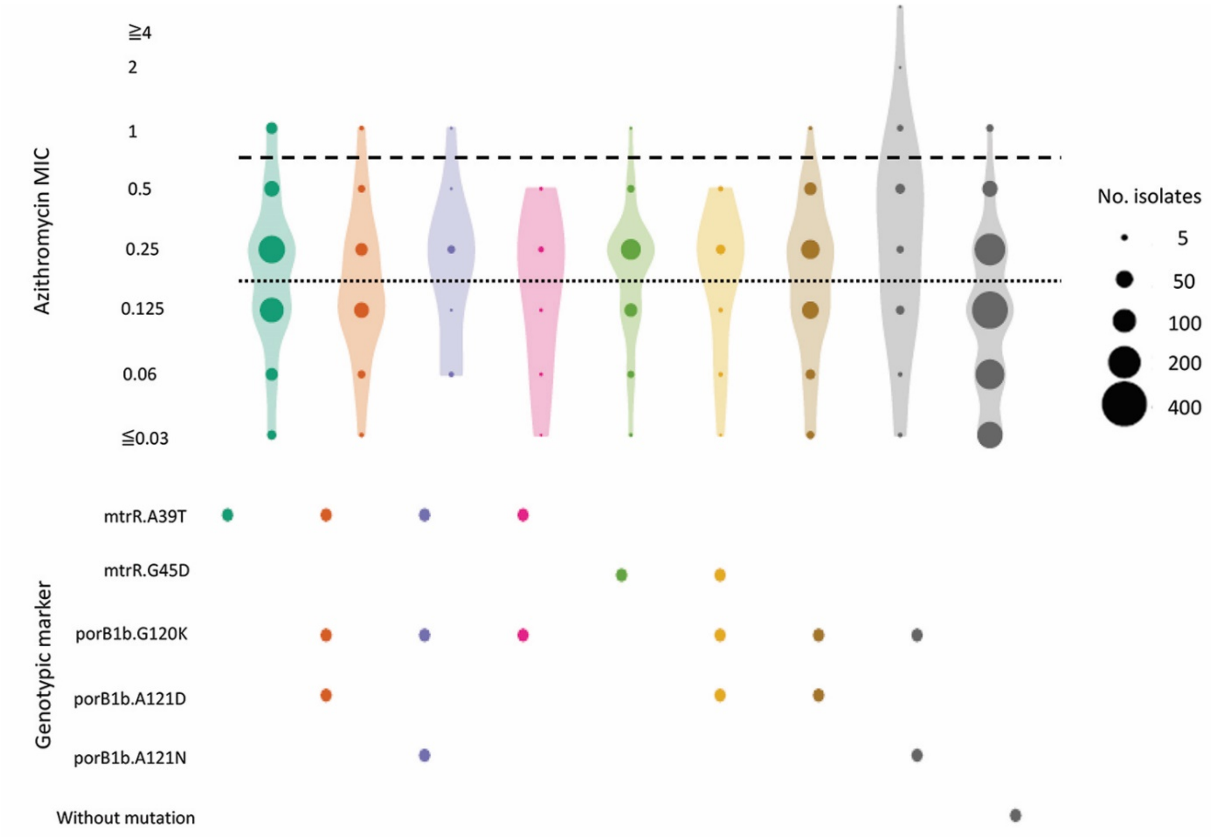

Appendix Figure 3. Genetic markers of antimicrobial resistance and MIC for azithromycin in a study of antimicrobial susceptibility of *Neisseria gonorrhoeae*, England, 2013–2016. Only marker combinations with ≥5 isolates included, dotted line indicates reduced susceptibility threshold of MIC ≥0.25 mg/L. Dashed line indicates resistance threshold of MIC >0.5 mg/L. Colors are a visual aide to distinguish every 4 groups of genotypic markers and are not representative of any genotypic types. AMR, antimicrobial resistance; MIC, minimal inhibitory concentration.

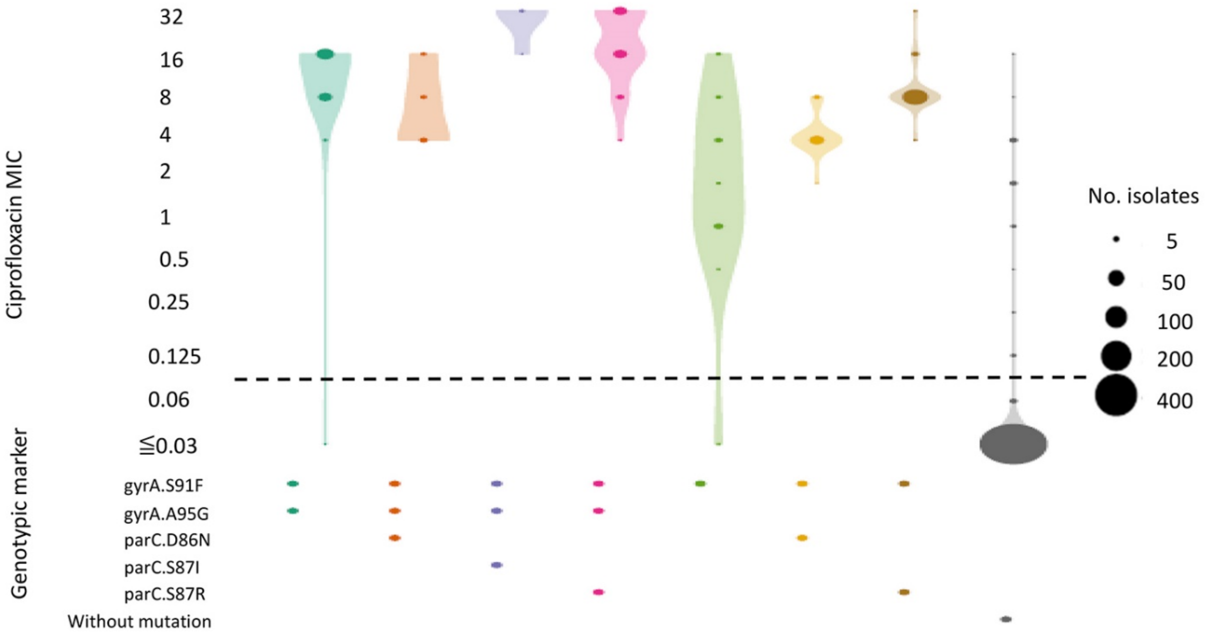

Appendix Figure 4. Genetic markers of antimicrobial resistance and MIC for ciprofloxacin in a study of antimicrobial susceptibility of *Neisseria gonorrhoeae*, England, 2013–2016. Only marker combinations with >5 isolates included. Dashed line indicates resistance threshold of MIC >0.06 mg/L. The colors are a visual aide to distinguish every 4 groups of genotypic markers and are not representative of any genotypic types. For isolates from 2016, only data on whether the isolate was susceptible or resistant to ciprofloxacin were available, so for MIC analyses all resistant isolates were allocated an MIC of 1 mg/L and all sensitive isolates were allocated an MIC of <0.03 mg/L. AMR, antimicrobial resistance; MIC, minimal inhibitory concentration.

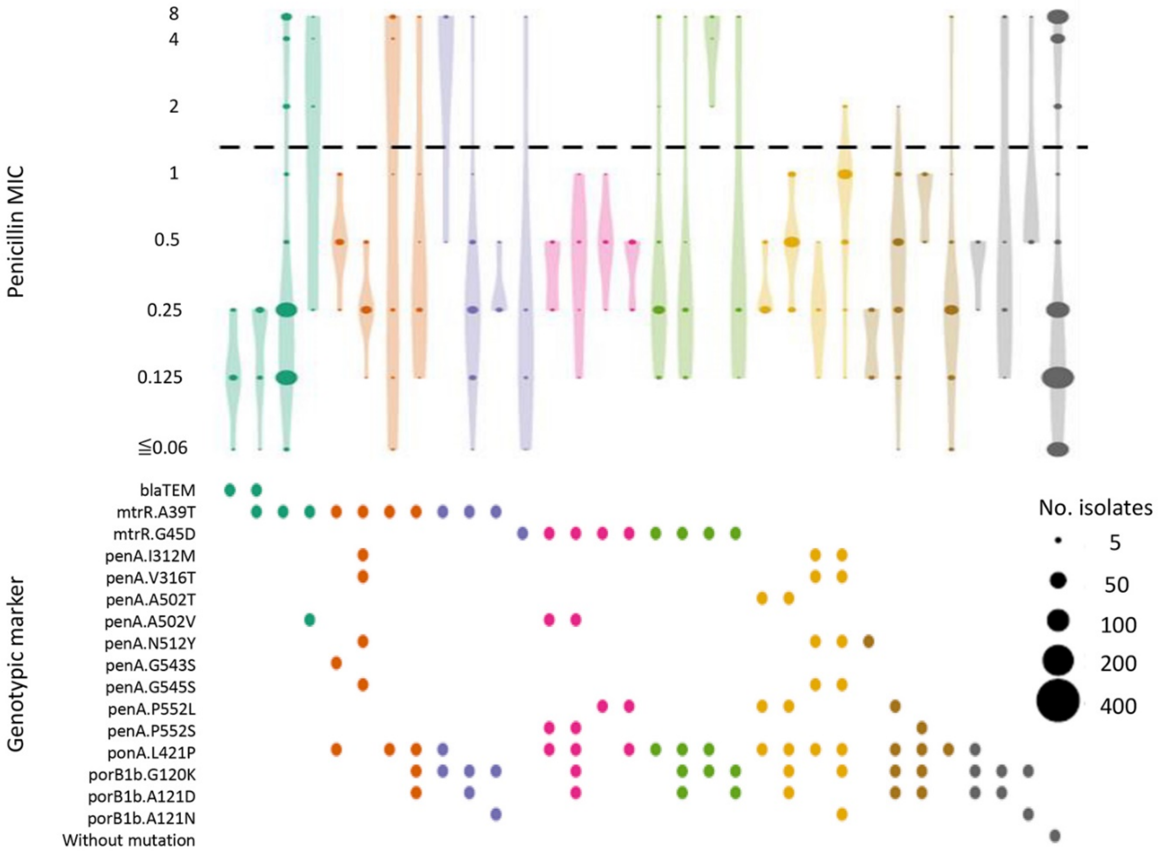

Appendix Figure 5. Genetic markers of antimicrobial resistance and MIC for penicillin in a study of antimicrobial susceptibility of *Neisseria gonorrhoeae*, England, 2013–2016. Only marker combinations with ≥5 isolates included. Dashed line indicates resistance threshold of MIC ≥1 mg/L. The colors are a visual aide to distinguish every 4 groups of genotypic markers and are not representative of any genotypic types. AMR, antimicrobial resistance; MIC, minimal inhibitory concentration.
